# W-band frequency selective digital metasurface using active learning-based binary optimization

**DOI:** 10.1515/nanoph-2024-0628

**Published:** 2025-02-07

**Authors:** Young-Bin Kim, Jaehyeon Park, Jun-Young Kim, Seok-Beom Seo, Sun-Kyung Kim, Eungkyu Lee

**Affiliations:** Department of Applied Physics, Kyung Hee University, Yongin-Si, Gyeonggi-Do, 17104, Republic of Korea; Department of Electronic Engineering, Kyung Hee University, Yongin-Si, Gyeonggi-Do, 17104, Republic of Korea

**Keywords:** digital metasurface, frequency selective surface, active learning, binary optimization

## Abstract

The W-band is essential for applications like high-resolution imaging and advanced monitoring systems, but high-frequency signal attenuation leads to poor signal-to-noise ratios, posing challenges for compact and multi-channel systems. This necessitates distinct frequency selective surfaces (FSS) on a single substrate, a complex task due to inherent substrate resonance modes. In this study, we use a digital metasurface platform to design W-band FSS on a glass substrate, optimized through binary optimization assisted by active learning. The digital metasurface is composed of a periodic array of sub-wavelength unit cells, each containing hundreds of metal or dielectric pixels that act as binary states. By utilizing a machine learning model, we apply active learning-aided binary optimization to determine the optimal binary state configurations for a given target FSS profile. Specifically, we identify optimal designs for distinct FSS on a conventional glass substrate, with transmittance peaks at 79.3 GHz and Q-factors of 32.7.

## 1 Introduction and results

The W-band, spanning frequencies from 75 to 110 GHz, is particularly noted for its applications in advanced radar systems, such as automotive collision avoidance and weather monitoring [1], [2], [3], high-resolution imaging for security screening [4], and communication systems requiring large bandwidth capacities, such as satellite communications [5] and high-speed data links [6], [7]. A notable issue associated with utilizing the W-band is the rapid attenuation of signals in the atmosphere, primarily due to absorption and scattering inherent to its high-frequency nature [8], [9]. This problem becomes even more difficult as W-band applications increasingly demand compact designs and the simultaneous use of multiple frequencies, which necessitate a multi-layer radio frequency (RF) design to accommodate different frequency bands. Such designs can limit the effective cross-sectional area available for signal detection due to the added complexity and potential interference between layers [10], [11]. To address these obstacles, a promising solution involves integrating frequency selective surfaces (FSS) on a single substrate, where different areas are optimized for specific frequencies [12], [13], [14]. This approach allows the substrate to handle multiple frequencies effectively, optimizing transmission and enhancing the signal-to-noise ratio, thus improving the precision and reliability of signal detection and communication within the system.

**Figure 1:**
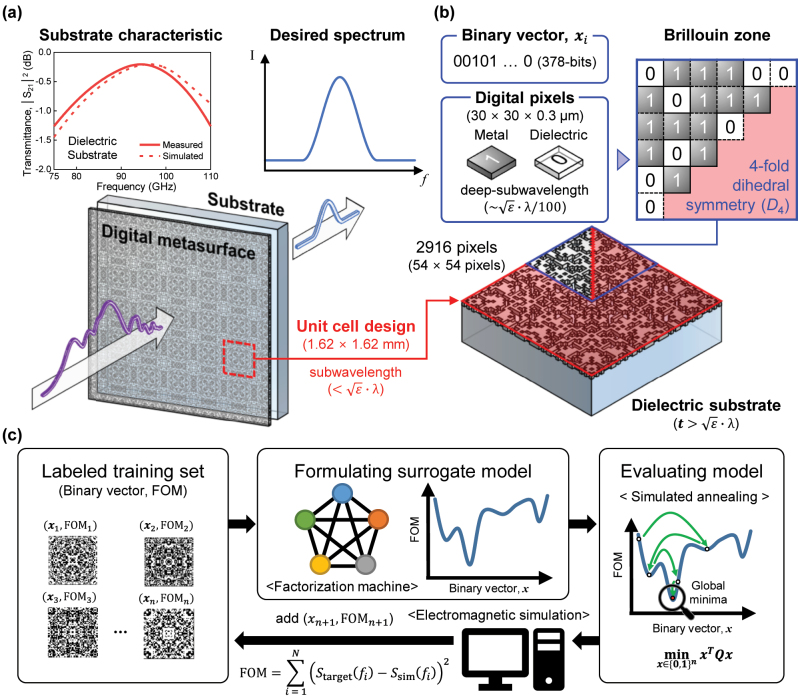
Design scheme of frequency selective surface (FSS) using digital metasurfaces with active learning-based binary optimization. (a) Schematic illustration of a digital metasurface performing FSS. (Top left) The inset shows the measured and calculated transmittance of the dielectric substrate used in our experiment, which supports Fabry–Perot resonances within the W-band frequency regime. (b) Sub-wavelength unit cell of the digital metasurface that consists of 2,916 (54 × 54) pixels. The pixels are encoded as 378 bits, with each bit representing a deep sub-wavelength 30 × 30 μm pixel: ‘1’ indicates a metal (silver) pixel, while ‘0’ indicates a dielectric (air) pixel. (c) Schematic of the digital metasurface optimization process using active learning. Details of each step are described in the main text.

In designing distinct FSS on a single substrate, a key challenge emerges from Fabry–Perot resonance, which is influenced by the thickness and dielectric constant of the substrate. For example, a 700-micron-thick glass substrate can exhibit a transmission peak at 93 GHz within the W-band frequency range, reflecting typical FSS behavior (see Figure 1(a)). This inherent resonance complicates the design process by potentially interfering with the target frequency, presenting an obstacle. To address these issues, metasurfaces can be utilized, as they offer enhanced control over the phase and wavefront of electromagnetic waves at the target frequency through metal periodic patterns on a sub-wavelength scale. However, at W-band frequencies, the conventional approach of using metasurfaces based on predetermined shapes, such as ring resonators or inductive-capacitive circuit patterns, faces limitations. At higher frequencies, transmission line or equivalent circuit-based design become less accurate due to the increased absorption and substrate interferences. The resonance behavior of these traditional designs may not scale linearly to such high frequencies. The challenge is further complicated when incorporating distinct FSS on a single substrate for multiple channel frequencies, as this approach may require intensive optimization to design metasurfaces for each of specific frequencies to counteract the inherent resonance of the substrate. This highlights the need for a platform with greater design freedom and flexibility to address the complexities at high frequencies.

A digital metasurface, with a unit cell composed of 10^2^ to 10^3^ deep sub-wavelength pixels (e.g., approximately 1/100 of the wavelength), has been studied for 5G applications and designed for various functional films [15], [16], [17], [18], [19], [20], [21], such as radomes [15], microwave energy harvesting [16], polarization converter [18], [19], and 5G antennas [20], [21], [22]. Each pixel in the unit cell can be in either a metal or dielectric material, representing a binary state. Thus, designing a digital metasurface is a binary optimization problem, where finding an optimal binary vector to achieve a target objective (e.g., a transmittance profile) involves exploring an extremely large space of 2^*N*^ total candidates. As this binary optimization is an ultra-high dimensional problem with *N* typically ranging from 10^2^ to 10^3^, it needs highly efficient algorithms. To date, heuristic optimization methods such as discrete particle swarm optimization [15], [16], genetic algorithms [17], [18], [19], and conformational space annealing [20], [21] have been employed to tackle this challenge, highlighting the need for even more efficient algorithms [22]. Recently, active learning-based binary optimization has been proposed to optimize nanophotonic structures and solid-state materials [23], [24], [25], [26], [27], [28], [29], [30], [31], [32], [33]. This approach uses a machine learning model to construct a surrogate function that approximates the binary optimization task, facilitating the efficient identification of optimal configurations. By iteratively building a sparse training dataset of binary vectors and their associated figures of merit (FOM), this method more effectively explores the hypervolume near the optimal solution, thereby enhancing computational efficiency and design outcomes. Although active learning has not yet been applied to the optimization of digital metasurfaces, it can offer a promising strategy for improving computational efficiency in this area.

In this paper, we studied the use of a digital metasurface as a platform for designing distinct FSS on a single substrate tailored for the W-band frequency regime. The design task was conducted using active learning-assisted binary optimization, which employed a second-order factorization machine (FM) to formulate a surrogate function that was then optimized by simulated annealing (SA) [34], [35], [36]. The unit cell of the digital metasurface was designed with eight-fold rotational symmetry relative to the optical axis of the incident electromagnetic wave and consisted of several hundred deep sub-wavelength pixels (e.g., *N* = 378) arranged in a non-repeatable zone. As representative cases, we successfully identified optimal pixel configurations for digital metasurfaces that functioned as efficient FSS at two distinct operational windows – one spanning 79–81 GHz and the other from 93 to 95 GHz – thereby overcoming the inherent resonance of the substrate. We analyzed the electromagnetic properties of the optimized digital metasurface, considering surface impedance matching for non-locality and surface current density for local effect. Furthermore, we fabricated the designed digital metasurfaces using conventional thin-film patterning processes and verified their FSS characteristics within the W-band frequency regime using a vector network analyzer.

The digital metasurface is designed on a conventional substrate to function as an FSS with a target function (i.e., *T*_target_), which represents the desired electromagnetic response through an optimized digital pixel configuration. In our case, this function corresponds to the transmittance spectrum that represented by the scattering parameter *S*_21_. The optimal design should overshadow the inherent resonance mode of the substrate. We transform the design task into an active learning-based binary optimization as follows. The digital metasurface features a square unit cell with a side length of 1.62 mm, divided into 54 × 54 square pixels (e.g., each with a side length of 30 μm). Since each pixel is approximately 1/90 of the wavelength (2.7 mm at 110 GHz), the digital metasurface can act as a single optical thin-film to manipulate the wave profile (e.g., phase and amplitude) of incoming electromagnetic waves in the W-band spectrum. The unit cell has two-dimensional 4-fold dihedral symmetry (*D*_4_), with both 4-fold rotational and 4-fold reflection symmetry about its central axis, yielding reduced Brillouin zone contains 378 pixels (see Figure 1(b)). This symmetric constraint was adopted to minimize polarization dependance and enhance computational efficiency by shrinking the binary search space from 2^2,916^ to 2^378^ during the optimization process. Each pixel is in either a ‘metal’ or ‘dielectric’ state, encoded as a binary ‘0’ or ‘1’, forming a binary vector of length 378. This configuration, defined by a specific binary vector (*x*_*i*_), characterizes a digital metasurface with a transmittance spectrum (i.e., *T*_*i*_), yielding a FOM_*i*_ for *x*_*i*_, defined as:

(1) $$\text{FO}{\text{M}}_{i}=\overset{f_{\max}}{\underset{f_{\min}}{\int }}{\left[T_{\text{target}}\left(f\right)-T_{i}\left(f\right)\right]}^{2}\text{d}f,$$

where *f*_min_ and *f*_max_ are the minimum and maximum frequencies of the operational window of the FSS. The transmittance spectrum, $T_{i}\left(f\right)$ , can be evaluated by solving Maxwell’s equations using a finite element method (e.g., CST Studio Suite). In this scheme, discovering an optimal design of the digital metasurface that satisfies *T*_target_ can be considered a binary optimization to identify an optimal binary vector that minimizes FOM.

**Figure 2:**
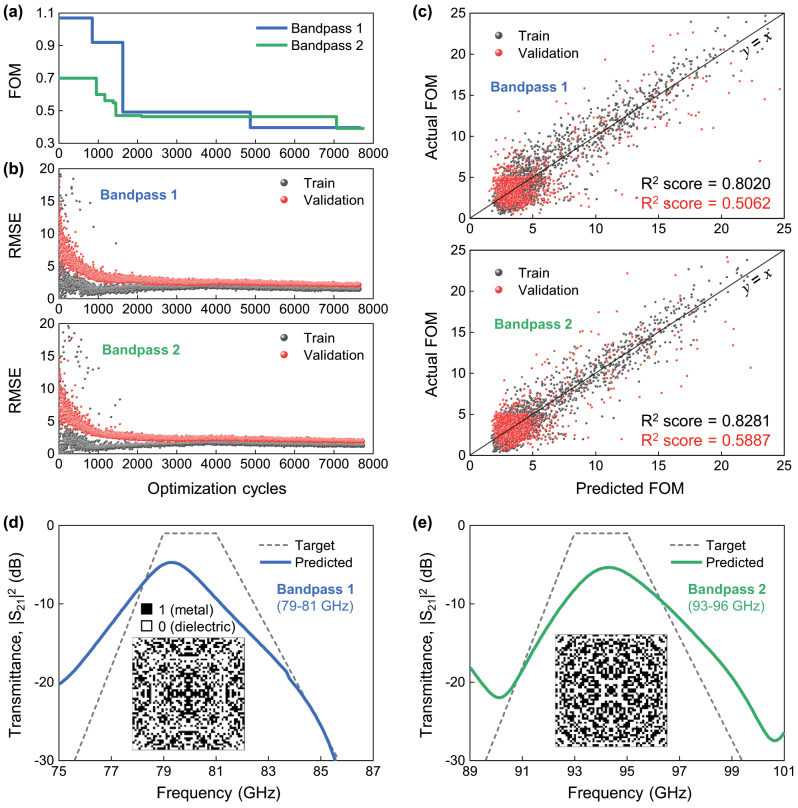
The performance of the active learning-based optimization for FSS using the digital metasurface. (a) The minimum FoM values in the accumulated training dataset as a function of optimization cycles for Bandpass 1 and Bandpass 2 cases. (b) Root-mean-square-error (RMSE) values as a function of the optimization cycles for the training set and validation set. (c) Parity plot for the training set and validation set with *R* ^2^ scores (black for the training set and red for the validation set). (d and e) Target and optimized transmittance spectra for (d) Bandpass 1 and (e) Bandpass 2. In (d) and (e), the trapezoidal gray dashed line indicates the target spectra. The inset images display the pixel configuration of the unit cell of optimized digital structures at 7,600 cycles, where black and white areas indicate metal and dielectric pixels, respectively.

For binary vectors with lengths smaller than fifty (∼2^50^), previous studies have shown that active learning effectively navigates the *N*-dimensional hypervolume of binary space near the optimal point with a sparse training dataset, efficiently identifying optimal binary vectors. However, designing a digital metasurface involves ultra-high dimensional binary optimization (∼2^378^), raising the question of whether active learning remains effective. Our study demonstrates that active learning can still manage such high-dimensional optimization tasks (as will be shown in Figure 2). Active learning consists of five steps (see Figure 1(c)): (i) preparing an initial training dataset (*X*_*n*_) consisting of *x*_*i*_ and FOM_*i*_: $X_{n}=\left\{\left(x_{1},\text{FO}{\text{M}}_{1}\right),\left(x_{2},\text{FO}{\text{M}}_{2}\right),\ldots ,\left(x_{n},\text{FO}{\text{M}}_{n}\right)\right\}$ ; (ii) formulating an FM-based surrogate function with the training dataset; (iii) optimizing the surrogate function using simulated annealing (SA) to identify a candidate binary vector; (iv) obtaining an FOM_*_ of the candidate binary vector (*x*_∗_) by solving Maxwell’s equations using the finite element method; (v) adding the candidate binary vector and its associated FOM to the training dataset, updating it to $X_{n+1}=\left\{X_{n},\left(x_{*},\text{FO}{\text{M}}_{*}\right)\right\}$ . These steps form an ‘optimization cycle’. Active learning continues until the number of optimization cycles reaches a predefined stopping criterion. Further details about active learning-based binary optimization can be found in the References [24], [25], [26], [27], [28], [29] and Methods sections.

To demonstrate the versatility of the digital metasurface designed through active learning, we define *T*_target_, as a trapezoidal functions:

(2) $$T_{\text{target}}\left(f\right)=\left\{\begin{array}{cc} \frac{T_{\max}-T_{\min}}{f_{L}-f_{A}}\left(f-f_{A}\right)+T_{\min}, & f_{A}\leq f<f_{L},\\ T_{\max}, & f_{L}\leq f\leq f_{H},\\ \frac{T_{\max}-T_{\min}}{f_{H}-f_{B}}\left(f-f_{B}\right)+T_{\min}, & f_{H}<f\leq f_{B}, \end{array}\right.$$

where *T*_max_ and *T*_min_ are the maximum transmittance in the passband and the minimum transmittance in the stopband, set to −1 and −30 dB, respectively. These values, corresponding to 80 % and 0.1 % power transmission, are commonly used in free-space applications to minimize signal interference, particularly in short-range radar systems such as those in autonomous vehicles. *f*_*L*_ and *f*_*H*_ denote the lower and upper side-band frequencies of the passband. *f*_*A*_ and *f*_*B*_ are the boundary frequencies of the operational window, determining the rate of transition between the passband and stopband. We applied active learning to two distinct bandpass cases with center frequencies at 80 GHz and 94 GHz, referred to as Bandpass 1 and 2, respectively. For the Bandpass 1, *f*_*L*_, *f*_*H*_, *f*_*A*_, and *f*_*B*_ are set to 79, 81, 75.6, and 85.7 GHz, and the corresponding values for the Bandpass 2 are 93, 95, 89.6, and 99.4 GHz, respectively.

In our active learning approach, the initial training dataset contains 25 binary vectors and their associated FOMs. The learning process is halted after 7,800 optimization cycles. We plot the minimum FOM value from the training dataset against the number of optimization cycles (see Figure 2(a)). Notably, active learning identifies moderate optimal binary vectors for both targets around 1,500 optimization cycles, achieving FOMs of 0.49 and 0.47 for Bandpass 1 and Bandpass 2, respectively. Extending optimization to 7,600 cycles results in further optimized structures, with FOMs improving to 0.40 for Bandpass 1 and 0.39 for Bandpass 2. These results align with previous studies involving binary vectors of smaller lengths, demonstrating the versatility of active learning-based optimization [24], [25], [26], [27], [28], [29]. Interestingly, at 7,600 optimization cycles, the training dataset represents only a minuscule fraction (∼10^−110^) of the total possible binary vectors (2^378^ dimensions equal ∼10^113^ combinations), highlighting the sparsity of the dataset. Despite this, the root-mean-square-error (RMSE) for both the training dataset and validation set converges and become similar (though not exactly the same) at 7,600 cycles (see Figure 2(b)). This indicates that the FM-based surrogate function effectively captures the hypervolume of the binary space. This is further supported by parity plots in Figure 2(c). The *R* ^2^ score, which measures the deviation of the prediction of the surrogate function from actual observations, ranges from 0.5 to 0.58, indicating moderate prediction accuracy.

We believe that the identified optimal binary vectors in active learning may not represent the global optimum. Nevertheless, the digital metasurfaces defined by these binary vectors exhibit promising FSS characteristics. For example, in the Bandpass 1 (or 2) scenario, the transmittance peaks at 79.3 GHz (or 94.3 GHz) with a Q-factor of 32.7 (or 32.1), which closely matches the targeted transmittance profile with a Q-factor of 33.5. These characteristics differ significantly from the inherent resonance spectrum of the substrate, suggesting that the interaction between incident electromagnetic waves and the identified digital metasurface dominates the inherent resonance. Yet, it remains challenging to discern which interactions are correlated with the digital metasurface design, as the unit cells, shown in the insets of Figure 2(d) and (e), display non-intuitive patterns that conventional circuit models may struggle to account for. Moreover, the distinct differences between the unit cell patterns of Bandpass 1 and Bandpass 2 emphasize the complexity and capacity to potentially produce unique electromagnetic responses inherent in the digital metasurface design.

**Figure 3:**
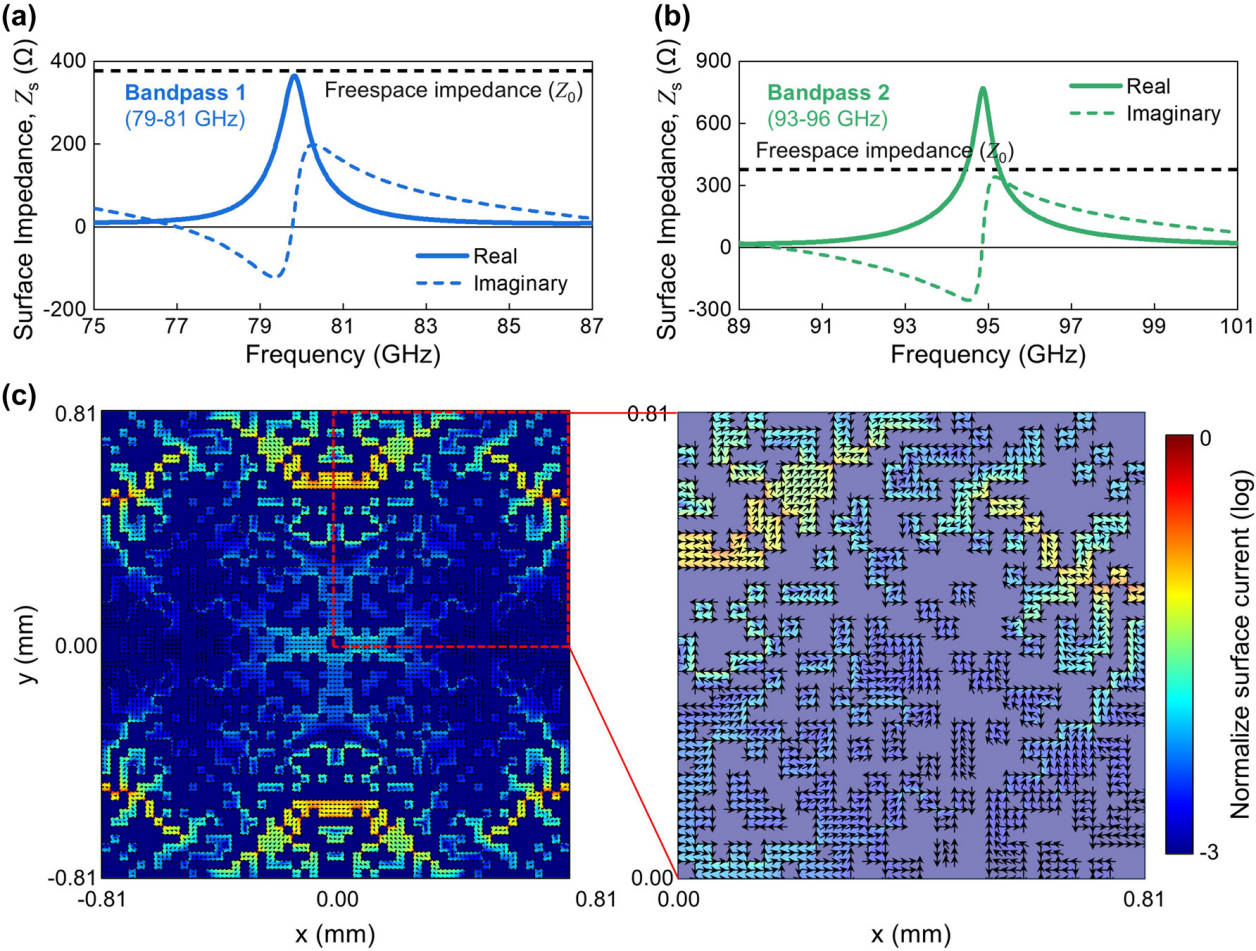
Electromagnetic analysis of the optimized pixel configuration of digital metasurfaces. (a and b) The calculated surface impedance (*Z*_*s*_) of the digital metasurfaces for (a) Bandpass 1 and (b) Bandpass 2 as a function of W-band frequencies. (c) The calculated surface current density distribution at the digital air/metasurface/substrate interface for the Bandpass 1 configuration at normal incidence (*θ* = 0°) of *E*_*x*_ polarization at 80 GHz. The right panel is a magnification of the square zone highlighted by the red-dotted line in the left panel. The color indicates the magnitude of the normalized surface current density on a logarithmic scale, and the black arrows represent the direction of the surface current density.

To clarify the interaction mechanism of the digital metasurface, we calculated the surface impedance at the metasurface/substrate interfaces from the simulated S-parameters (see Figure 3(a) and (b)) [37]. To decouple the metasurface from substrate modes, the substrate was modeled as effectively infinite in thickness to eliminate back-reflections (see Methods). In both the Bandpass 1 and Bandpass 2 cases, a pronounced peak in the real part of the impedance was observed near 80 GHz and 94 GHz, respectively, which matches the transmittance peak of Bandpass 1 and Bandpass 2 shown in Figure 2(d) and (e). For Bandpass 1, the real part value of the surface impedance is very close to the freespace impedance at the peak frequency. For Bandpass 2, there are crossing points between the freespace impedance and the real part of the surface impedance spectrum near the peak. This analysis suggests that the incident electromagnetic wave can pass with high transmittance across the digital metasurfaces by minimizing the offset of the impedance with respect to the free-space near the peak, indicating that the digital metasurface can function as a single optical element. Meanwhile, to further elucidate the working mechanism, we studied the surface current density at the metasurface/substrate interface at the resonance frequency. The normalized magnitude and direction of the surface current density were obtained by taking the difference between tangential H-fields across the interface, which are shown in Figure 3(c). In the unit cell of Bandpass 1 at 80 GHz, distinct regions of high-density currents are observed in the peripheral zones, showing localized interactions with the incident electromagnetic wave. The induced surface current collectively forms circulating patterns attributed to the symmetrical arrangement, which are very similar to capacitive-inductive resonances. These results show that the digital metasurface can support localized interactions, as well as the substrate resonance modes.

**Figure 4:**
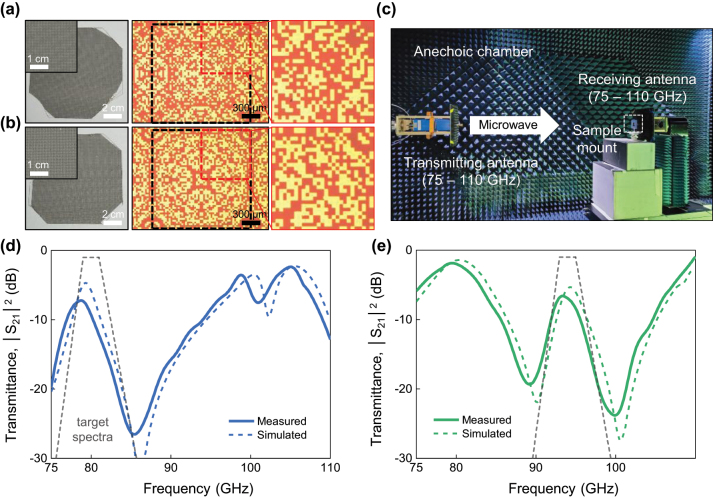
Experimental verification of the optimized Bandpass 1 and Bandpass 2 digital metasurfaces. (a and b) (Left) Digital photo and (Right) reflective-optical-microscope images of the fabricated 300 nm-thick Ag digital metasurfaces for (a) Bandpass 1 and (b) Bandpass 2 configurations. In (a) and (b), yellow (or orange) regions depict metal (or dielectric) pixels, and the black-dashed boxes indicate the unit cells. (c) Digital photo image of the experimental system to measure *S* parameters of digital metasurfaces in an anechoic chamber. (d and e) Measured W-band transmittance spectra of (d) Bandpass 1 and (e) Bandpass 2 digital metasurfaces. The dashed lines are calculated spectra of the optimized structures for the W-band range, and the trapezoidal gray dashed lines represent their target spectra used in the active learning process.

We fabricated the designed digital metasurfaces, Bandpass 1 and 2, on a large glass substrate (10 cm × 10 cm, thickness of 0.7 mm) using conventional thin-film patterning process, which includes photolithography and e-beam evaporation process. The digital metasurface is evenly fabricated over the glass substrate, confirmed by digital photo images in Figure 4(a) and (b). Also, optical microscope images reveal the fabricated metasurfaces have distinctable pixel configurations with fine edges, successfully realizing the optimal digital metasurface designed by the active learning. The transmittance spectra of the fabricated digital metasurfaces were obtained using a vector network analyzer in an anechoic chamber (see Figure 4(c)). In the target spectra region, the peak of measured spectra is 78.7 GHz with −7.2 dB for Bandpass 1 or 93.4 GHz with −6.6 dB for Bandpass 2, which is slightly lower frequency with minor reduced transmittance in respect to the simulated spectra (see Figure 4(d) and (e)). Outside of the target spectra region, the measured spectra have another valleys and peaks, which are also slightly shifted to the lower frequencies in compared with those of the simulated spectra. For example, the measured spectra of Bandpass 1 shows two valleys at 85.4 GHz and 101.0 GHz and two peaks at 98.8 GHz and 105.0 GHz. The measured spectra of Bandpass 2 exhibits two valleys 89.2 GHz and 99.9 GHz and one peak at 79.5 GHz. These peaks and valleys come from the fact that their frequency regime was not included in the FOM definition. As a result, the optimization was solely focused on achieving the desired bandpass performance within the target spectral range. To achieve a flatter spectrum and minimize peaks and valleys outside the passband, the target spectra can be redefined to incorporate stricter spectral constraints, such as setting the out-of-passband region to −30 dB. But, overall, the shape of measured transmittance spectra of the fabricated metasurfaces are very similar to those of simulated spectra, demonstrating that the versality of digital metasurface for realization.

## 2 Conclusion

In conclusion, we identified the optimal design of digital metasurfaces for two different FSSs, Bandpass 1 and Bandpass 2, on a glass substrate using active learning-assisted binary optimization. Although the hypervolume of the binary space is extremely large, active learning exploiting an FM-based surrogate function and successfully discovered optimal pixel configurations within the reduced zone, which are not intuitive. The impedance matching condition could explain the role of the digital metasurface at the peak of transmittance, suggesting it works as a single optical element that reduce the impedance mismatching between the digital metasurface to the freespace. Meanwhile, the analysis with surface current density revealed a critical cluster of metal pixels strongly interacting with the electromagnetic wave, highlighting the existence of a local effect. However, the relationship between the local effect and the non-local phase shifting has not yet been unfolded, which may be challenging due to the complexity of pixel configurations. Further exploration of reducing the unit cell and pixel size could provide deeper insights into the underlying mechanisms of the pixelated configuration and potentially improve the performance of frequency-selective filters. The platform of digital metasurfaces can be leveraged for various applications in the W-band, such as beamforming and beam steering, and is also applicable to other microwave frequency ranges. Additionally, the deep-subwavelength structures can be readily fabricated using standard microfabrication techniques, such as conventional semiconductor fabrication or printed circuit board (PCB) manufacturing techniques, to ensure cost-effective and scalable large-area implementations.

## 3 Methods

### 3.1 Active-learning-based binary optimization

We used an open-source FM package, xLearn, to formulate a surrogate function in active learning and used an SA sampler implemented in the D-wave Ocean package to optimize the FM-based surrogate function. The process began by generating an initial binary dataset of 25 randomly sampled binary vectors along with their corresponding figure-of-merit (FOM) values. These FOM values, representing the performance of each digitized metasurface configuration, were computed through finite element method (FEM)-based electromagnetic simulations using commercial software (CST Studio Suite, Simulia). The initial dataset was split into a training set (80 %, 20 vectors) and a validation set (20 %, 5 vectors). The FM model was trained to capture the relationship between the input binary vector ***x*** (defining the structure) and the output *y* (FOM), as described by the following objective function:

$$y:=w_{0}+\overset{n}{\underset{i=1}{\sum }}w_{i}x_{i}+\frac{1}{2}\overset{k}{\underset{j=1}{\sum }}\left[{\left(\overset{n}{\underset{i=1}{\sum }}v_{ij}x_{i}\right)}^{2}-\overset{n}{\underset{i=1}{\sum }}{v_{ij}}^{2}x_{i}^{2}\right],$$

where *w*_0_, *w*_1_, and *v*_*ij*_ denoted the bias term, linear coefficient, and quadratic coefficient, respectively. The latent factor size *k* of the FM model was set to 16. Since this objective function can be represented as a Hamiltonian in a quadratic unconstrained binary optimization (QUBO) model, SA can efficiently search for the optimal binary vector. The QUBO Hamiltonian is expressed as follows:

$$y=\underset{x\in {\left\{0,1\right\}}^{n}}{\sum }x^{T}Qx.$$

In the training, the FM model may exhibit limited accuracy due to the sparse dataset, so the identified optimal binary vector *x*_∗_ may not represent the true global optimum. To address this, we computed the actual FOM of *x*_∗_ through electromagnetic simulation and added this result to the dataset. If *x*_∗_ was already in the dataset, a new random binary vector was generated, its FOM evaluated, and the data incorporated. The FM model was then retrained with the updated dataset, and the optimization cycle was restarted.

### 3.2 Electromagnetic simulation

Electromagnetic simulations for active learning were conducted using commercial software (CST Studio Suite, Simulia) with a frequency-domain finite element method (FEM) to compute transmission and reflection coefficients. The simulation domain was defined from 0 to 0.81 mm along both the *x* and *y* axes, oriented perpendicular to the wave propagation direction. Only a quarter of the unit cell was simulated, with perfect electric conductor (PEC) boundaries applied normal to the *x*-axis and perfect magnetic conductor (PMC) boundaries normal to the *y*-axis. Along the *z*-axis, a perfectly matched layer (PML) boundary condition was implemented, with an additional quarter-wavelength freespace buffer to ensure substrate resonance effects. Waveguide ports along the *z*-axis, configured with a fundamental *E*_*x*_-polarized mode, were employed to approximate plane wave incidence in free space. The permittivity of the glass substrate (Eagle-XG) was set to experimentally measured values of 4.95 + 0.05*i*. Silver was modeled using a surface impedance approach with a conductivity of 6.3 × 10^7^ S/m, suitable for high frequencies where the skin effect confines fields to a minimal depth, optimizing computational efficiency while accurately capturing conductive properties.

### 3.3 Surface impedance estimation

Electromagnetic simulations were conducted to extract the effective surface impedance at the metasurface/substrate interface. The simulation framework was adapted from the previously described S-parameter analysis, with specific modification to mitigate the Fabry–Pérot resonances. To decouple the metasurface from substrate modes, the substrate was modeled as effectively infinite in thickness, removing the buffer layer and employing perfectly matched layer (PML) boundary conditions at the end to eliminate back-reflections. Additionally, a phase de-embedding technique was employed to exclude the contributions from freespace and substrate propagation, isolating the intrinsic metasurface response at the interface. The effective surface impedance was determined from the simulated S-parameters using following expression [33]:

$$Z_{\text{s,eff}}=Z_{0}\sqrt{\frac{{\left(1+S_{11}\right)}^{2}-{S_{21}}^{2}}{{\left(1-S_{11}\right)}^{2}-{S_{21}}^{2}}}=Z_{0}\sqrt{\frac{\mu_{\text{eff}}}{\varepsilon_{\text{eff}}},}$$

where *S*_11_ and *S*_21_ are the complex reflection and transmission coefficients, respectively, and *Z*_0_ = 377 Ω is the freespace impedance.

### 3.4 Fabrication of digital metasurfaces

Digital metasurfaces were fabricated on glass substrates (Eagle-XG) via standard semiconductor processing techniques, including photolithography and vacuum deposition. A 2 µm-thick layer of negative photoresist (DNR-L300-D1) was spin-coated onto the glass substrate, followed by ultraviolet (UV) exposure through chrome photomasks using a mask aligner (MA-150e, Karl Suss). Subsequently, a 300 nm-thick Ag layer was deposited via electron beam evaporation (UEE, ULTEC). The Ag layer was patterned by lift-off in acetone solution, completing the metasurface fabrication.

### 3.5 W-band S-parameters measurement

To measure transmission coefficients *S*_21_, we used a vector network analyzer (E8362C, Agilent Technologies) equipped with millimeter-wave VNA extenders (V10VNA2-T/R, V10VNA2-T, OML). WR-10 horn antennas served as both the transmission and reception antennas for the millimeter-wave signals. Fabricated samples were mounted on a custom sample holder, fabricated with a 3D printer (310 F, Cubicon). All spectral measurements were carried out inside an anechoic microwave chamber.
